# Hypergravity-induced changes in actin response of breast cancer cells to natural killer cells

**DOI:** 10.1038/s41598-021-86799-7

**Published:** 2021-03-31

**Authors:** Minseon Lee, Dongjoo Kim, Soonjo Kwon

**Affiliations:** 1 Department of Biological Sciences and Bioengineering, Inha University, 100 Inharo Nam-gu, Inchon, 22212 South Korea; 2 Biology and Medical Device Evaluation Team, Korea Testing and Research Institute, Gwacheon, Korea

**Keywords:** Cancer therapy, Immunotherapy, Regenerative medicine, Tissue engineering

## Abstract

Although immunotherapy holds promising cytotoxic activity against lymphoma or leukemia, the immunosuppressive mechanisms of solid tumors remain challenging. In this study, we developed and applied a hypergravity exposure system as a novel strategy to improve the responsiveness of breast cancer cells to natural killer (NK) cells for efficient immunotherapy. Following exposure to hypergravity, either in the presence or absence of NK cells, we investigated for changes in the cell cytoskeletal structure, which is related to the F-actin mediated immune evasion mechanism (referred to as “actin response”) of cancer cells. Breast cancer cell line MDA-MB-231 cells were exposed thrice to a 20 min hypergravitational condition (10 × g), with a 20 min rest period between each exposure. The applied hypergravity induces changes in the intracellular cytoskeleton structure without decreasing the cell viability but increasing the cytotoxicity of MDA-MB-231 from 4 to 18% (4.5-fold) at a 3:1 ratio (NK-to-target). Analyses related to F-actin further demonstrate that the applied hypergravity results in rearrangement of the cytoskeleton, leading to inhibition of the actin response of MDA-MB-231. Taken together, our results suggest that the mechanical load increases through application of hypergravity, which potentially improves efficiency of cell-based immunotherapies by sensitizing tumors to immune cell-mediated cytotoxicity.

## Introduction

NK cells are lymphocytes that play a pivotal role in innate immunity and respond rapidly to transfections or tumor cells ^1^. The activating receptor NKG2D on the surface of the NK cell recognizes and responds to substances lacking the MHC class I chain-related gene A/B (MICA/B) as foreign substances (“missing self” recognition) ^2,3^. Recently, CAR-NK, designed similarly to CAR-T, has been developed to express the chimeric antigen receptor (CAR) without recognition by a deficiency of MHC class I ^4^. In addition, genetic modification was applied to enhance cytotoxicity by selecting specific target markers only for certain cancers such as B-cell malignancy (CD19) ^5^, acute myeloid leukemia (CD33) ^6^, and multiple myeloma (MM, CS1) ^7^. Furthermore, studies are being conducted to increase antibody-dependent cell-mediated cytotoxicity, and improve the therapeutic effect by combination therapy with currently developed monoclonal antibodies ^8^.

Immune cell-based cancer therapy has emerged as a major breakthrough in cancer research. However, cell-mediated immunotherapy remains challenging because the immune activity of NK cells is often inhibited in the tumor microenvironment ^9^. Hypoxic conditions surrounding cancer cells inhibit the upregulation of major activating NK cell receptors such as NKp46, NKp30, NKp44, and NKG2D ^10^. Also, transforming growth factor-β (TGF-β) released by cancer cells is a major immunosuppressive cytokine, which inhibits anti-tumor activity of immune cells ^11^. Furthermore, mechanisms that directly evade immunity of NK cells have been discovered in some cancer cell lines. PD-L1, expressed specifically in malignant tumors (including in multiple myeloma (MM)), plays an important role in tumor-induced immune suppression (immune checkpoint) ^12^. In particular, in the triple-negative human breast cancer (TNBC) cell line MDA-MB-231, there is evidence that the intracellular cytoskeleton of the tumor plays an important role in immune resistance to NK cells. At an immunological synapse in contact with NK cells, cancer cells are resistant to lysed granules such as granzyme B or perforin by “actin response”, where the restructuring of F-actin occurs temporarily ^13^. The present study focuses on the proposed cytoskeleton-mediated immune evasion mechanism. We designed a system that increases the mechanical load of cells, as an innovative strategy to improve NK cell-mediated cytotoxicity by inhibiting such immune evasion. This system attempts to modify the cytoskeleton structure of the cell by applying a mechanical load increase to suppress “actin response”, an immune evasion mechanism mediated by F-actin. A previous study had established a hypergravity environmental exposure system and confirmed that hypergravity affects the cellular skeletal structure in cells ^14^. Thus, we intended to increase NK cell-mediated cytotoxicity by applying mechanical load increase under hypergravity, and inducing changes in the intracellular structure in the cells. We identified the effect of the mechanical load increase by applied hypergravity on cell viability, NK cell-mediated cytotoxicity, cytoskeletal structure, and related gene expressions. By increasing the mechanical load suppressing the immune evasion mechanism of the target cancer cells (MDA-MB-231), we established the method improving responsiveness of target tumor cells (actin response positive MDA-MB-231) to the immune cells (NK cells) for efficient immunotherapy, not increasing the killing capacity of NK cells themselves in this study.

## Results

### Effects of hypergravity on cytotoxicity of breast cancer cells

**Figure 1:**
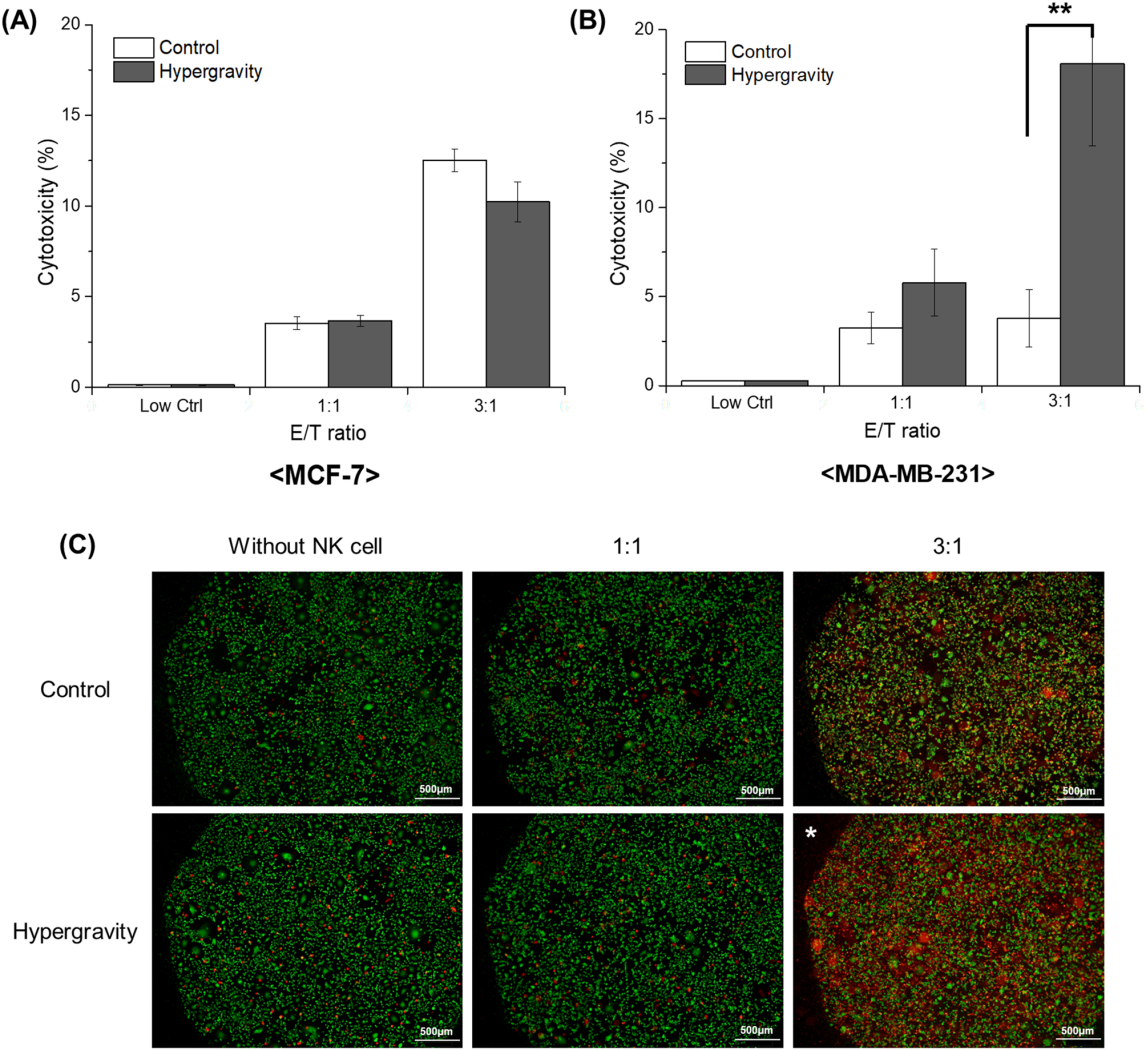
In Vitro LDH cytotoxicity assay of NK cells. LDH cytotoxicity assay data in (**A**) MCF-7, an actin response-negative cell line, and (**B**) MDA-MB-231, an actin response-positive cell line (n = 3). (**C**) Live/dead assay imaging of MDA-MB-231 cells was carried out to confirm the effects of hypergravity on NK cell-mediated cytotoxicity. Each live and dead cell is displayed in green (stained with calcein AM) and red fluorescence (stained with ethidium-1), respectively. Hypergravity stimulation induces significant increase only in cytotoxicity of MDA-MB-231 in the presence of NK cells, as compared to the control group without hypergravity exposure.

We first identified effects of the hyper-gravitational condition itself on viability of target cancer cells, either in the presence or absence of NK cells. Target cancer cells cultured in 6-well plates were exposed to NK cells at two different effector:target (E:T) ratios for 4 h, as described in Materials and Methods. Immediately after 4 h subsequent to incubation of target cancer cells with NK cells under hypergravitational condition, cell viability and cytotoxic activity of NK cells were evaluated with LDH cytotoxicity assay. In both MCF-7 cells (Fig. 1A) and MDA-MB-231 cells (Fig. 1B,C) without NK cell treatment, there was no significant difference in cell viability between control and test group, thereby indicating that cell viability of target cancer cells is not affected by the hypergravity condition itself.

MCF-7, an actin response-negative cell line, showed higher cytotoxicity in the presence of more NK cells, but no significant difference was observed in the cytotoxicity under both hypergravity and no hypergravity conditions at all E:T ratio. Conversely, with increased number of NK cells, MDA-MB-231, a cancer cell line with evasive mechanisms by actin response, showed less cytotoxicity without hypergravity, but significantly higher cytotoxicity under conditions of hypergravity. Especially with a ratio of 3:1 E:T, the cytotoxicity exerted by NK cells significant increases from 4 to 18%. Similar to the LDH assay results (Fig. 1B), the live/dead cytotoxicity assay also showed that cytotoxicity of MDA-MB-231 significantly increases in the hypergravity-exposed group (Fig. 1C).

### Effects of hypergravitational conditions on actin cytoskeletal structures

To check the effect of hypergravity on intracellular cytoskeletons, MDA-MB-231 cells were divided into four groups: control group (no NK cells and no hypergravity), hypergravity-treated group without NK cells, NK cell-treated group without hypergravity, and NK cell-treated group with hypergravity.

**Figure 2:**
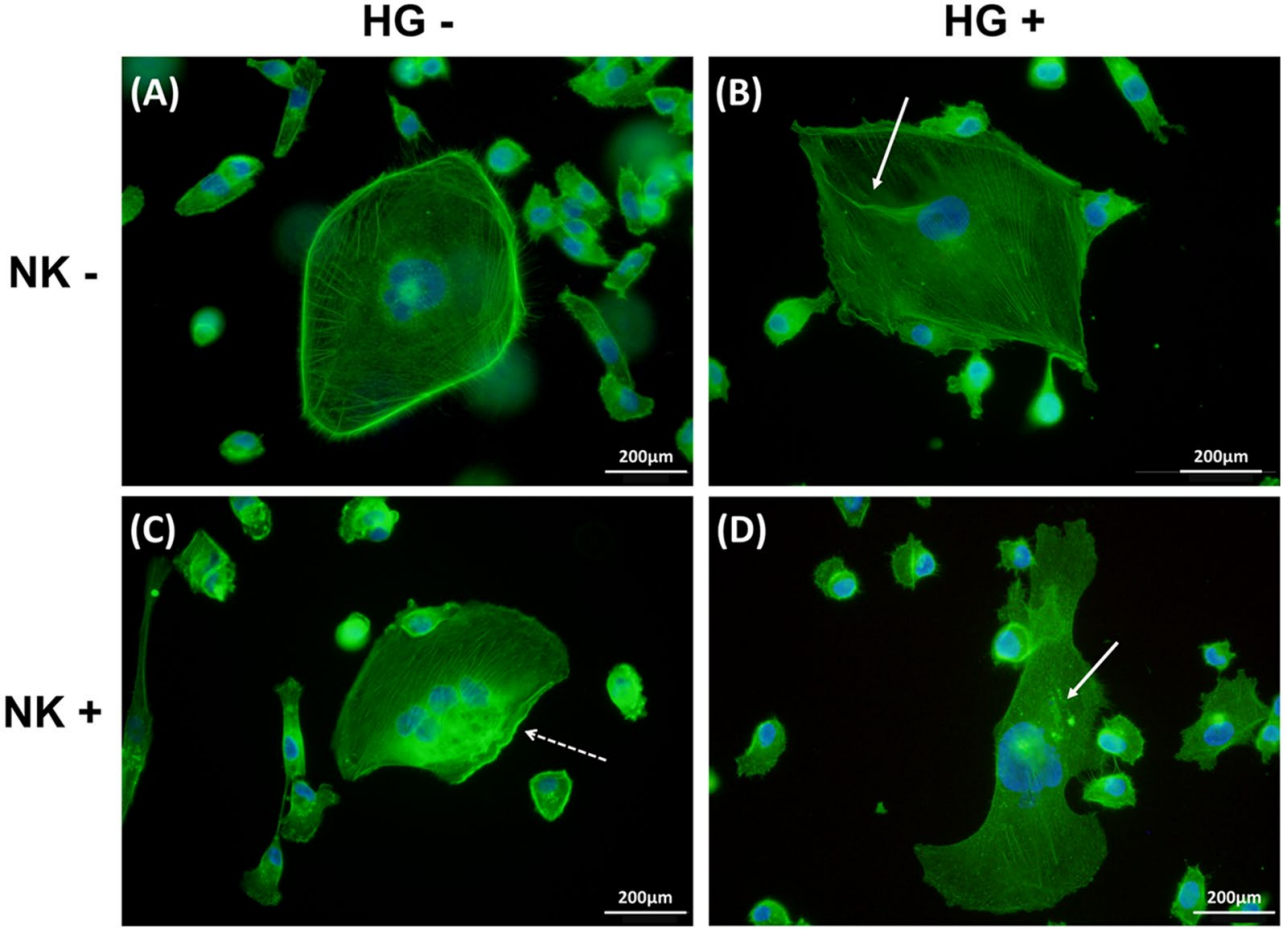
F-actin immunofluorescence in MDA-MB-231. Immunofluorescence images of F-actin with AlexaFluor 488 conjugated Phalloidin. Nucleus is counterstained with DAPI. (**A**) control group in MDA-MB-231 without hypergravity (HG) and without NK cells; (**B**) MDA-MB-231 cells exposed to hypergravity and without NK cells; (**C**) MDA-MB-231 cells without hypergravity and with NK cells; (**D**) MDA-MB-231 exposed to both hypergravity and NK cells. Changes in the cytoskeletal structure are indicated with white solid arrow, and actin response with white dotted arrow.

As seen in the control group (Fig. 2A, Supplementary Fig. 2A–C), the cellular skeletal structure is relatively evenly distributed, and the linear structure of filamentous F-actin persists. However, in the group exposed to hypergravity (Fig. 2B, Supplementary Fig. 2D–F), the intracellular cytosol shows greater distortion and dotted structure (indicated by white arrow) than the filamentous linear structure, and density of the skeletal structure is lesser than the control group. Especially, the F-actin structure was combined, forming a large void (Fig. 2B). Thus, compared to the control group, we confirmed that changes appear in the F-actin structure under conditions of hypergravity.

In fluorescence stained images of the NK cells treated group (Fig. 2C, Supplementary Fig. 2G–I), the actin structure appears more clear only near the cell membrane (white dotted arrow) at the immunological synapse formed by the contact between MDA-MB-231 and NK cells. In contrast with NK cell-treated group without hypergravity, fluorescence images of MDA-MB-231 in the presence of NK cells treated with hypergravity (Fig. 2D, Supplementary Fig. 2J–L) appeared lower rate of actin response in the presence of and less dense actin structure due to mechanical load by hypergravity.

Therefore, our results confirm that structural changes in the actin cytoskeletal structure are induced by external stimuli such as hypergravity or NK cells.

### Degradation of F-actin cytoskeleton under conditions of hypergravity

**Figure 3:**
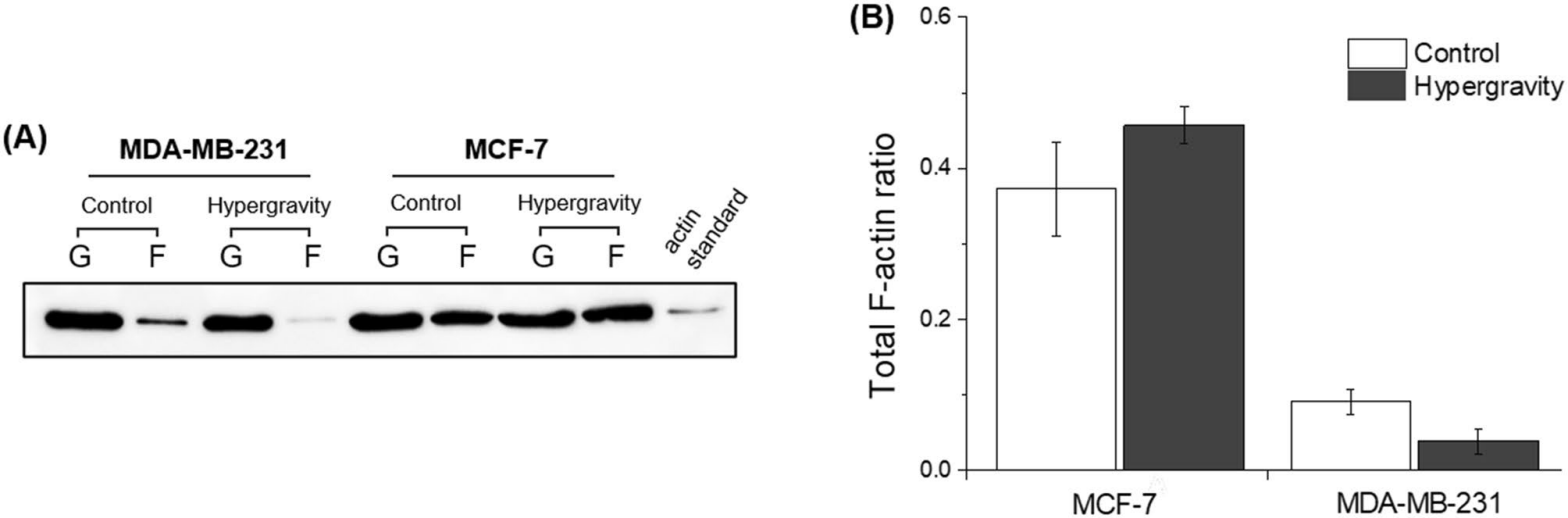
Western blot analysis of F-actin/G-actin. (**A**) Western blot showing the effects of hypergravity on the ratio of F-actin (expressed as F-)/total actin (F + G). (**B**) Graph of total F-actin ratio in static control group and hypergravity exposed group.

To analyze the remaining portion of F-actin in the total intracellular actin, Western blot was performed on F-actin and G-actin intracellular proteins. The ratio of total F-actin (F-actin versus total actin) was apparently reduced under hypergravitational condition. Reduction of the total F-actin ratio was observed only in actin response-positive cells, viz., MDA-MB-231 (Fig. 3A). Total F-actin ratio was analyzed by Syngene GeneTools software (Fig. 3B). MCF-7 under hypergravitational condition showed a slight increase in total F-actin ratio, which was not statistically significant. In comparison, the total F-actin ratio of MDA-MB-231 under hypergravity reduced from 9 to 4%.

### Upregulation of genes related to F-actin polymerization

**Figure 4:**
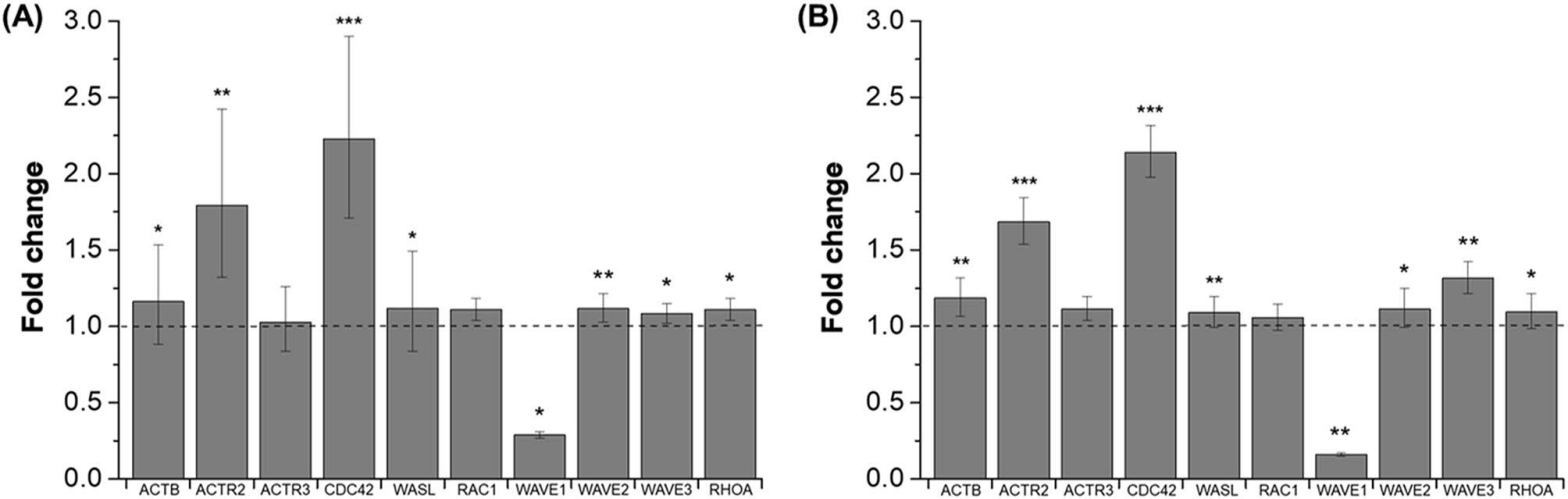
Fold changes in gene expression levels of proteins related to F-actin polymerization in BCa cells. Fold changes in mRNA level following exposure to hypergravity in MCF-7 (**A**), and in MDA-MB-231 (**B**) cells. Housekeeping gene GAPDH and the static control (n = 3) were also analyzed by RT-qPCR.

Gene expression levels of ACTB, ACTR2/3, CDC42, WASL, RAC1, WAVE1/2/3, and RHOA were determined by performing RT-qPCR (Fig. 4). Increased gene expressions of these F-actin formation related factors (except WAVE 1) were observed subsequent to hypergravity exposure, with most increases being statistically significant. The CDC42 expression level increased by 120% in MCF-7 and 114% in MDA-MB-231 (*P* ≤ 0.001). Level of ACTR2 was also statistically increased by 79% in MCF-7 (*P* ≤ 0.01) and 68% in MDA-MB-231 (*P* ≤ 0.001), whereas changes were not significant in ACTR3 and RAC1 expressions.

However, only the expression level of WAVE1 was observed to statistically decrease by 72% in MCF-7 and 84% in MDA-MB-231, which will be discussed further.

## Discussion

Breast cancer (BC) is one of the most commonly diagnosed cancers, with 2.1 million new cases reported globally in 2018 ^15^. In the United States (US) alone, over 3.8 million women were reported with a history of BC in 2019 ^16^. The TNBC subtype lacking expression of the estrogen receptor (ER), progesterone receptor (PR), and human epidermal growth factor receptor-2 (HER-2), accounts for approximately 15–20% of all BC cases, and is insensitive (unresponsive) to the usual hormone therapies ^17^. Although immunotherapy has emerged as a new breakthrough^18^, the immune evasion mechanism of BC is challenging, since it hampers achieving the desired therapeutic effects ^19^. Immunosuppressive factors need to be considered as the major targets in future studies, to broaden successful immunotherapeutic strategies ^20^.

Among the various immune suppressive mechanisms in play, temporary remodeling of the cytoskeleton structure of cells plays an important role. Cytoskeleton is the key to NK cell's recognition of the target, forming and working on an immunological synapse ^21^. In this regard, it is known that mechanical stress through vibration, or the hypergravity exposure system established through prior research, affects the cytoskeletal structure in cells ^14,22^. Therefore, based on the results of previous studies, changing the skeletal structure of cells to improve the cytotoxic activity of immune cells is a potential new strategy. In the current study, we hypothesize that changes in the cytoskeleton induced by mechanical load increase affects the interaction between tumor cells and NK cells at the immunological synapse by suppressing the immune suppressive mechanisms. Therefore, as a strategy to improve the efficiency of immune cell-based therapy, we designed a system to increase the mechanical load applied through hypergravity.

Based on the results of the LDH assay in this study and other previous researches ^23,24^, moderate hypergravity (10 × g) does not affect cell viability. In particular, there was no change in viability of the actin response negative MCF-7 cell line following exposure to hypergravity with subsequent NK cell treatment (Fig. 1A). However, hypergravity exposure affected the actin response positive MDA-MB-231 cells subjected to NK cell treatment. In other words, cytotoxicity of MDA-MB-231 cells significantly increased in the presence of NK cells following exposure to hypergravity (Fig. 1B). This result was further supported by the live/dead assay of MDA-MB-231 and MCF-7 cells in the presence of NK cells following exposure to hypergravity (Supplementary Fig. 2). Taken together, our results confirm that mechanical load increase by hypergravitational condition induces preferred changes in improving the cytotoxic effects of NK cells only in the actin response positive cell line, which has resistance through intracellular cytoskeleton remodeling. We, therefore, established a relationship between hypergravity and actin response, and it is thought that the increased mechanical load applied through gravity inhibits the occurrence rate of the actin response, in the actin response positive MDA-MB-231 cells.

We specifically demonstrated the effects of hypergravity on the cytoskeleton structure through immunofluorescence imaging. Following exposure of hypergravity, the F-actin cytoskeleton reduced in density and the structures were often interrupted by voids (Fig. 2B,D), while cells in the control group possessed a well-organized F-actin structure. Versari et al. reported that HUVEC cells grown in hypergravitational conditions show disassembly of actin fibers which tend to accumulate at the periphery of cells near the plasma membrane ^25^. Consequently, it seemed certain that variation of gravitational condition induces alterations in cells, which is supported by Western blot data in the current study (Fig. 3). Conversely, hypergravity did not induce any significant alteration of the cytoskeleton in MCF-7, which is presented in the supplementary figure (Supplementary Fig. 3).

In addition, fluorescence imaging confirmed that NK cells cause temporary actin cytoskeleton remodeling in MDA-MB-231 cancer cells, which appear as higher density F-actin near the plasma membrane in contact with NK cells (Fig. 2C, Supplementary Fig. 2I). This mechanism possibly confers resistance to the cancer cells against the immune activity, which has been reported in a previous study ^13^. Compared to the previous studies identifying through live images, our results indicate with some certainty that changes in the cytoskeleton structure are induced by NK cells.

Furthermore, immunostaining images showed that MCF-7 (Supplementary Fig. 3) also presented substantial multinucleated cells (MNCs), but were not as prominent as the triple-negative cell line. MDA-MB-231 (in Fig. 2) showed more giant cells [sometimes inferred as polyploid giant cancer cells (PGCCs)], which may contribute to growth, invasion, metastasis and chemoresistance by forming a hypoxic microenvironment and generating mesenchymal like phenotype in the triple-negative breast cancer cell line ^26,27^.

Consistent with our previous data, the effects of gravity were similarly identified in the results of Western blot assay. For groups exposed to hypergravity in MDA-MB-231 cell lines, the total intracellular F-actin ratio was reduced (Fig. 3B). This tendency could be paralleled by immunofluorescence imaging data (Fig. 2), in which the intracellular total F-actin ratio was reduced after exposure to mechanical load. Thus, we infer that the overall decrease in F-actin protein results in inhibiting immune avoidance of mesenchymal-like phenotype MDA-MB-231 cell ^28^. To substantiate the fluorescent staining results, we confirmed that the mechanical load by hypergravity actually caused a decrease in the F-actin level in cells. This inclination to decrease in actin proportion following hypergravity has also been reported previously ^25,29^ in their data of qualitative alterations.

**Figure 5:**
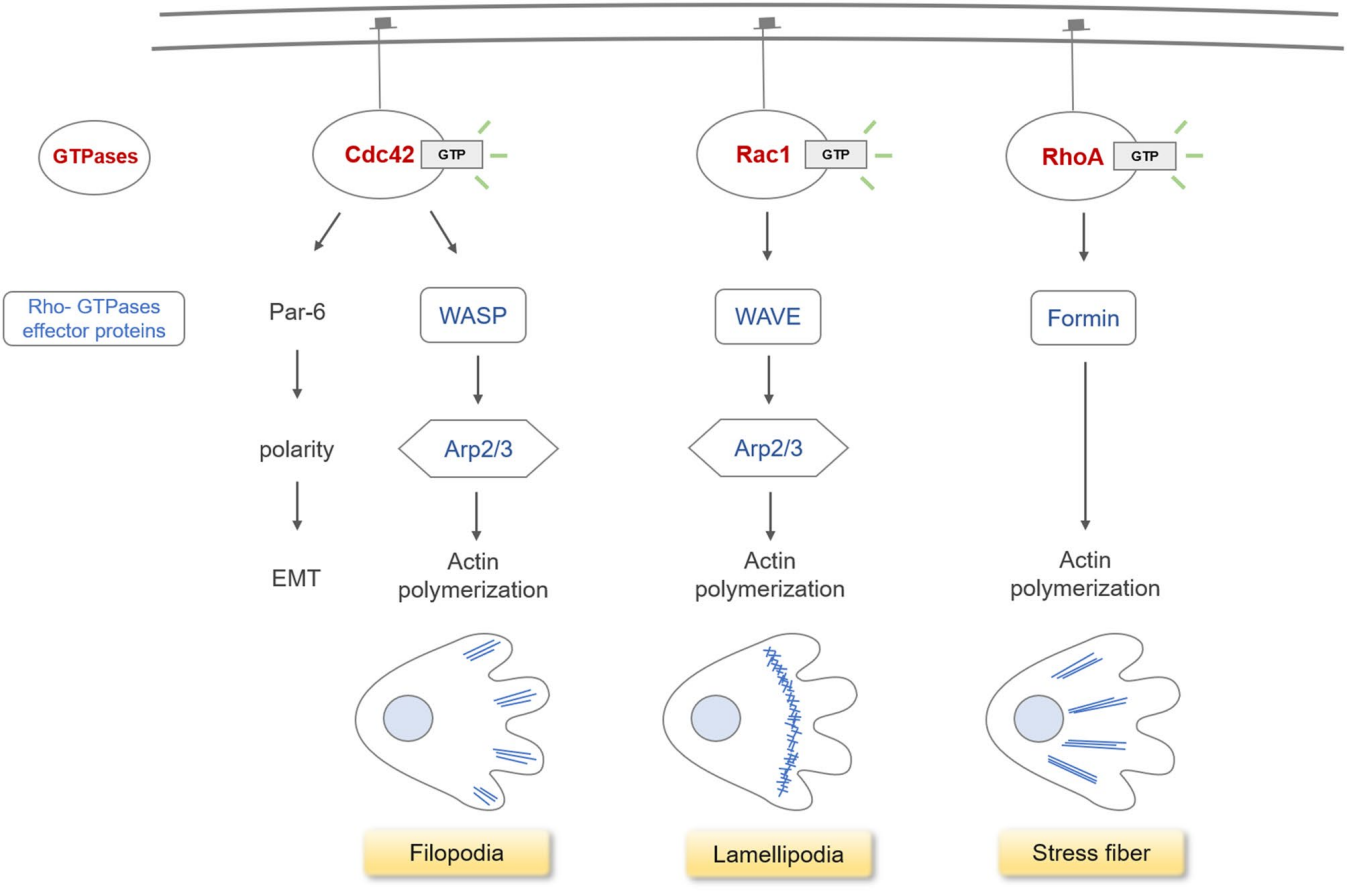
Formation pathway of cytoskeleton structure by F-actin. Filamentous actin is the major cytoskeletal protein in the cell, and there are three main structures formed by F-actin within a cell: filopodia, lamellipodia, and stress fiber. The Rho GTPases Cdc42, Rac1, RhoA are key elements in the regulation of actin filaments. Cdc42 and Rac1 induce actin polymerization through the WASP/WAVE family members, which are respectively involved in the formation of filopodia and lamellipodia by stimulating the Arp2/3 complex downstream. Stress fiber assembly is predominantly regulated by RhoA in the formin-mediated pathway.

During the process of hypergravity exposure, the protein level of intracellular F-actin decreased and there was a structural change (immunofluorescence, western blot data). After hypergravity exposure, the expression of the genes involved in F-actin formation has increased. The increased expression of related genes (such as WASL, CDC42, and ACTR2) observed in this study is thought to be an increase occurring after a temporary decrease in protein level of F-actin. It is well known that three main structures comprise F-actin within a cell: filopodia, lamellipodia, and stress fiber (Fig. 5). RT-qPCR was performed to determine if there were changes only in certain structures of F-actin. The markers selected for evaluation were β-actin (ACTB), small Rho family GTPase cell division control protein 42 homolog (CDC42), and N-Wiskott–Aldrich Syndrome protein (WASL) for the formation of filopodia; Ras-related C3 botulinum toxin substrate 1 (RAC1; a GTPase) and the Wiskott-Aldrich syndrome protein family Verprolin-homologous protein 1, 2, and 3 [WAVE1, 2 and 3, also known as and Wiskott–Aldrich Syndrome Protein Family Member (WASF)] for markers involved in the formation of lamellipodia; the Ras Homolog Family Member A (RHOA) acting as a GTPase as markers for the stress fiber construction process. In addition, the actin nucleating complex Arp2/3 (ACTR2, 3) was selected as a marker, in which WASL and WAVE in the previous two pathways interact and ultimately engage in the formation of F-actin.

Gene expressions were analyzed to investigate differences in expression levels of MCF-7 and MDA-MB-231 (Supplementary Fig. 4). ACTR3, CDC42, and RHOA expressions were higher in MDA-MB-231 than in MCF-7. In particular, N-WASP or CDC42 knockdown suggests that the filopodia structure formed by downstream effector ARP2/3 complex is responsible for the burst of actin polymerization and actin response following NK cell attack ^13^. Therefore, analysis of gene expression characteristics confirmed that the cause of difference in actin response frequency between the two cell lines is the difference in the expression levels of genes involved in filopodia formation (CDC42 and ACTR3).

Considering the effects of hypergravity, the expressions of all genes (except WAVE1) were observed to be increased in both cell lines (Fig. 4), and all changes (except ACTR3 and RAC1) were statistically significant. Compared with the Western blot analysis data, we inferred that expressions were increased to maintain F-actin at a certain level in the cells, which had reduced following exposure of mechanical load increase due to hypergravity. Of the three aforementioned F-actin structures, only the gene expression associated with a particular structure type did not increase significantly, but changes in expression of genes involved in the formation of filopodia were most significant.

Most gene expressions, except for WAVE 1, were increased to maintain consistency of the total ratio of F-actin structure decreased by mechanical load increase due to hypergravity, and to induce the reorganization of the cytoskeleton. WAVE 1 is not directly involved in the formation of the lamellipodial membrane protrusion, but plays an essential role in maintaining stability of the structure. Knock-down of WAVE1 reduced spread of the actin filament density to the periphery, with increased rate in formation of the curling edge ^30^. This seems to be somewhat related to immunofluorescence images of the hypergravity exposure groups, which show disassembly of the F-actin structure and the stress fiber in sparse quantity (Fig. 2B). Furthermore, in epithelial ovarian cancer (EOC), it is known that the WAVE 1 protein is involved in aggressiveness of tumor ^31^. Indeed, as a result of suppressing WAVE1 through mRNA interference, the cell migration, cell development, cell adhesion and cell proliferation of the tumor is decreased ^32^. Therefore, reduction in gene expression of WAVE1 following exposure to mechanical load increase by hypergravity provides a promising insight for therapeutic and prognostic potentials for tumors.

To conclude, the current study determines that mechanical load increase due to hypergravity increases cytotoxicity of tumors by inducing changes in the cytoskeletal structure. Tumors are resistant to NK cell attack, which is mediated by rapid actin remodeling in the response of this attack (“actin response”). Our experiments confirmed that exposure to a hypergravitational environment inhibits the immune avoidance mechanism, thereby making tumors vulnerable to immune attack. This result is supported by fluorescence imaging and Western blot analysis of F-actin, which show a decrease in total F-actin ratio. Gene expression levels of WASL, CDC42, and ACTR2, associated with F-actin polymerization, increase after exposure to reconstruct the cytoskeleton structure. These findings suggest the potential of hypergravity as a strategy for enhancing efficiency of NK cell-mediated cytotoxicity by improving responsiveness of target cells to immune cells, which can be used together with existing cancer therapies. In fact, exposing humans to 10 × g of hypergravity will probably induce a significant negative impact on health. Therefore, we should try to find other types of stimuli (eg, vibration, radiofrequency, or ultrasound) that can cause similar effects like hypergravity in the future. Our efforts in this study may help sensitize tumors to cell-based immunotherapies, thereby improving efficacy of the treatment.

## Materials and methods

### Materials

Human breast cancer cells MCF-7 and MDA-MB-231, Human Natural Killer effector cells NK-92MI, RPMI1640, FBS (Gibco), penicillin streptomycin (Gibco), α-MEM, horse serum (Gibco), sodium bicarbonate, inositol, 2-mercaptoethanol, folic acid, Dulbecco’s phosphate-buffered saline (DPBS; Gibco), LDH cytotoxicity assay kit (DoGenBio., DG-LDH500), CytoPainter Phalloidin-iFluor 488 reagent (ab176753, Abcam, Cambridge, UK), DAPI, formaldehyde, Triton X-100, G-actin/F-actin in vivo assay kit (Cytoskeleton, BK037), and mRNA primers.

### Cell culture

Human breast cancer cells MCF-7 and MDA-MB-231 were obtained from the Korean Cell Line Bank (KCLB; Seoul, Korea). The human Natural Killer effector cells NK-92MI were obtained from American Type Cell Collection (ATCC; Rockville, MD, USA). Breast cancer cells were cultured at 37 °C with 5% CO_2_, in RPMI1640 supplemented with 2 mM l-glutamine, 10% FBS (Gibco), and 1% penicillin streptomycin (Gibco). NK-92MI cells were cultured in α-MEM supplemented with 1.5 mM sodium bicarbonate, 0.2 mM inositol, 0.1 mM 2-mercaptoethanol, 0.02 mM folic acid, and 12.5% heat-inactivated FBS, and HS (Gibco) added in the final concentration. Since NK-92MI is an IL-2 independent cell line, fresh medium was added instead of giving complete medium change, maintaining an appropriate ratio of old and fresh medium.

### Hypergravity stimulation and NK effector cell treatment

Breast cancer cells cultured in 6-well plates were exposed thrice to hypergravitational condition (10 × g for 20 min) with 20 min interval between each exposure ^14^. After the hypergravity exposure, breast cancer cells were treated with NK effector cells in an Effector-to-Target (E:T) ratio of 1:1 and 3:1. NK cell-treated BCa cells were subsequently cultured in an incubator for 4 h. The live/dead cytotoxicity assay for determining cytotoxicity of breast cancer cells and F-actin immunofluorescence was performed after removing NK cells by giving three washes with DPBS. LDH cytotoxicity assay was conducted to measure viability of breast cancer cells using supernatant media component with each of control group (indicated below), after hypergravity or NK cell treatment.

### Cell viability

LDH cytotoxicity assay kit (DoGenBio., DG-LDH500) was used to measure viability of breast cancer cells after exposure to hypergravity (2 h) and NK cell treatment (4 h). Medium was collected from each well and centrifugated at 600*g* for 5 min, following which 10 μl supernatants in control and test groups were obtained and reacted with 100 μl LDH reaction mixture. Each control group such as media control, NK cell control (equal number of NK cells in each E:T ratio of test group) and high control was prepared following the assay kit protocol. After few minutes of reaction time in 96-well plates, absorbance was measured at 450 nm wavelength and NK cell-mediated cytotoxicity was calculated by applying the following equation through OD values, as described in the manufacturer’s protocol.

$$Cytotoxicity \left(\mathrm{\%}\right)=\frac{Exp. \,data-Target \,cell \,low \,control-Effector \,cell \,control}{Target \,cell \,high \,control-volume \,control} \times 100$$

### Live/dead cytotoxicity assay

Cell viability of BCa cells was determined using the LIVE/DEAD Viability/Cytotoxicity Kit for mammalian cells (L3224, Invitrogen, Carlsbad, CA, USA). Adherent MDA-MB-231 cells in 6-well plates were stained with 2 μM calcein AM and 4 μM ethidium homodimer-1 working solution after NK cell treatment (4 h) and washing 3 times with DPBS for removal of NK cells, and were analyzed by fluorescence microscopy (calcein AM; ex/em ~ 495 nm/ ~ 515 nm, EthD-1; ex/em ~ 495 nm/ ~ 635 nm), according to the manufacturer's protocol.

### F-actin immunofluorescence

AlexaFluor 488 conjugated phalloidin (ab176753, Abcam, Cambridge, UK) and DAPI were used for staining the F-actin cytoskeleton and nucleus, respectively. After removing culture medium, BC cells were washed twice with DPBS to remove dead cells debris or NK cells, followed by fixing with 4% formaldehyde (Sigma, St Louis, MO, USA) for 15 min. To increase permeability, 0.1% Triton X-100 was added to the fixed cells. Fixed BCa cells were subsequently incubated with phalloidin conjugate working solution in DPBS for 90 min, rinsed twice with DPBS, and mounted with mounting solution (Vectashield H-1200, Vector Laboratories, Burlingame, CA, USA) containing DAPI. Stained cells were observed using fluorescence microscopy at Ex/Em = 493/517 nm.

### Western blot

Total F-actin ratio was determined using the G-actin/F-actin In Vivo Assay Kit (BK037, Cytoskeleton, Denver, USA), following the manufacturer’s recommended protocol. Briefly, control and hypergravity treated BCa cells were treated with warm LAS2 lysis buffer for preparing the protein sample. Total lysates were pipetted and centrifuged at 2000 rpm for 5 min to pellet unbroken cells. After removing pellets, the supernatant was centrifugated at 100,000g for 1 h using ultracentrifuge (L-90K, SW 55Ti, Beckman Coulter, Brea, California, United States) to separate F-actin (present in the pellet fraction) from soluble G-actin (present in the supernatant fraction). Each actin protein sample was prepared by following the manufacturer’s protocol. Next, 10 μg sample of each protein sample was separated by electrophoresis through 12% sodium dodecyl sulfate–polyacrylamide gel, followed by transfer to a polyvinylidene difluoride membrane (162-0177, Bio-Rad, Hercules, CA, USA) using the semidry transfer method (Bio-Rad). Nonspecific binding was blocked using 5% skim milk in Tris-buffered saline for 1 h at room temperature. Membranes were subsequently incubated with primary antibodies against actin polyclonal antibody (AAN01, Cytoskeleton, Denver, USA), overnight at 4 °C. Probed membranes were then immersed in horseradish peroxidase conjugated secondary antibody (ab6721, Abcam) for 1 h at room temperature Blots were visualized by applying chemiluminescence reagents (W3651, GenDEPOT, Barker, TX, USA) and quantified using a chemiluminescence imaging system (G:BOX Chemi XRQ, Syngene, Cambridge, UK). Total F-actin ratio in cells was calculated in the ratio of F-actin versus total cellular actin (G-actin + F-actin).

$$Total \,F-actin\, ratio= \frac{F-actin}{Total \,actin \,(F-actin+G-actin)}$$

### Gene expression analysis

After exposing to 2 h hypergravity stimulation, total RNA of control and test group cells was extracted using the TRIzol reagent (Life Technologies, Carlsbad, CA, USA), following the protocol recommended by the manufacturer. A PrimeScript RT reagent kit (Takara, Shiga, Japan) was used to reverse transcribe 1 μg extracted mRNA to cDNA. The CFX96 detection system (Bio-Rad) was applied to detect the expression level of mRNA with TB Green Premix Ex Taq II (RR810A, Takara, Shiga, Japan). Target genes examined were β-actin (ACTB), Arp2/3 complex (ACTR2, 3), cell division control protein 42 (CDC42), N-Wiskott–Aldrich Syndrome protein (N-WASP, whose gene is referred to as WASL), Rac Family Small GTPase 1 (RAC1), Wiskott-Aldrich syndrome protein family Verprolin-homologous protein 1, 2, 3 (WAVE 1, 2, 3), and Ras Homolog Family Member A (RHOA). Expression levels of genes were determined by performing real-time quantitative PCR (RT-qPCR). Primer sequences for target genes are listed in Table 1. GAPDH was used as the housekeeping control gene.

**Table 1:** Primer sequences of target genes.

Target gene	Size (bp)	Sequences	Tm (°C)
GAPDH	120	F: GAAATCCCATCACCATCTTCCAGG	61.23
		R: GAGCCCCAGCCTTCTCCATG	62.62
ACTB	122	F: GTCATTCCAAATATGAGATGCGTTG	59.09
		R: TGCTATCACCTCCCCTGTGT	60.25
CDC42	112	F: GGCTGTCAAGTATGTGGAGTG	58.65
		R: CTTCCTTTTGGGTTGAGTTTCCG	60.24
WASL	123	F: ACACCAAGCAATTTCCAGCAC	59.93
		R: GTGTGCCTCTGAGATTCCACAC	61.19
ACTR2	73	F: CATCTTCCCAGCTTTGGTTGG	59.15
		R: ATCCTTGATTTCAATGTTTCCCAC	57.96
ACTR3	122	F: GCCTTAGCTGCATCTTGGACC	61.35
		R: CTGCCAATCACATACCCTTCAGC	61.80
RAC1	209	F: GGGAGACGGAGCTGTAGGTAA	60.68
		R: AGAACACATCTGTTTGCGGA	57.75
WAVE1	97	F: CCCTACCTGTAATCAGTGATGCC	60.50
		R: GCTTCCTGTTCACGCTGCTCTTA	63.26
WAVE2	185	F: GCAGCATTGGCTGTGTTGAAA	60.54
		R: CACACTGGATCTTTTGGGTCC	52.38
WAVE3	124	F: ACCGATGGCTCCAGCAGACTAC	64.06
		R: GCTGACGAAGGCAGTTTGTGC	62.90
RHOA	117	F: CTGTCCCAACGTGCCCATCA	62.75
		R: CTGCCTTCTTCAGGTTTCACCG	61.70

### Statistical analysis

All results in the experiments are expressed as mean ± standard error of mean values, and are representative of at least three independent experiments. A Student’s t-test in Microsoft Excel 2016 (two-tailed, equal variance) was used for determining the statistical significance (*P ≤ 0.05, **P ≤ 0.01 and ***≤ 0.001).

## Supplementary Information

Supplementary Information

## Data Availability

The authors declare that all the data supporting the finding of this study are available within the article and from the corresponding author on reasonable request.
