# Supplementary material for: Hypergravity-induced changes in actin response of breast cancer cells to natural killer cells
Source: Sci Rep. 2021 Mar 31;11:7267. doi: 10.1038/s41598-021-86799-7 (PMC8012622; doi:10.1038/s41598-021-86799-7)
Supplement: Supplementary file 1 — Supplementary Information [file 41598_2021_86799_MOESM1_ESM.docx]

**SUPPLEMENTARY MATERIAL**

**Hypergravity-induced changes in actin response of breast cancer cells to natural killer cells**

Minseon Lee^1^, Dongjoo Kim^2^, and Soonjo Kwon^1, *^

^1^ Department of Biological Engineering, Inha University, Incheon 22212, Korea

^2^ Biology and Medical Device Evaluation Team, Korea Testing & Research Institute,

Gwacheon, Korea

**
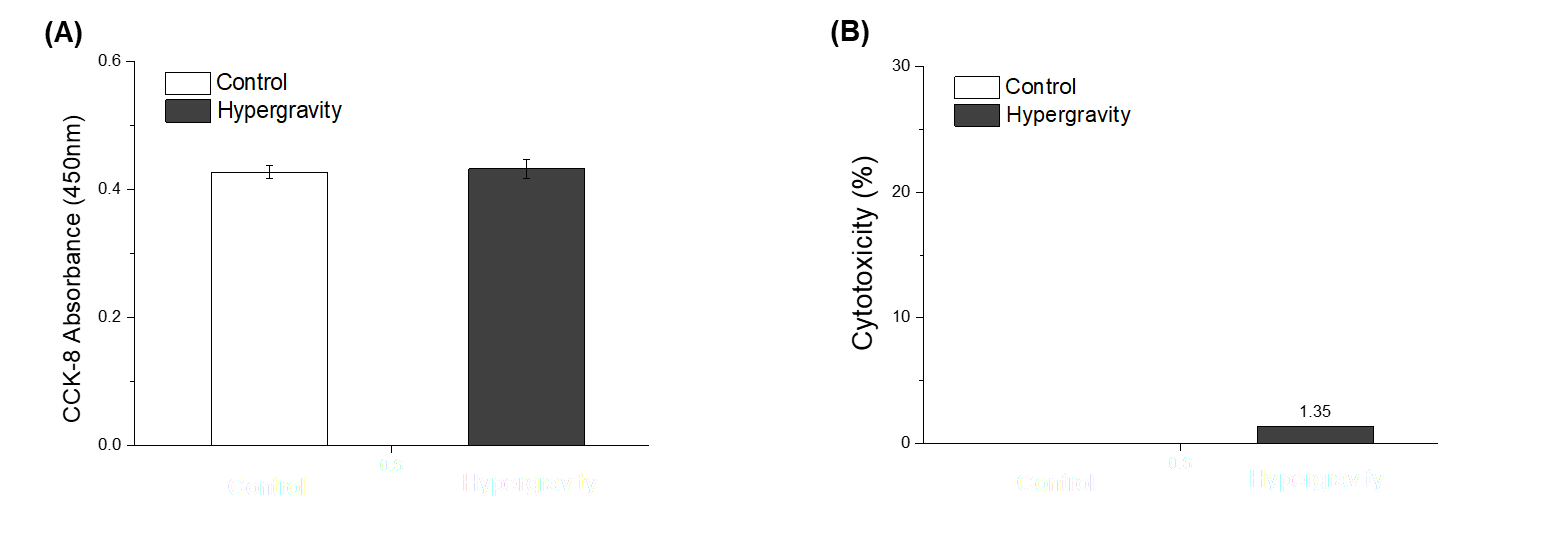
**

**Supplementary Figure 1.** Viability of NK-92MI following exposure to hypergravity. Cell viability was measured by (A) CCK-8 assay; (B) LDH cytotoxicity assay. The applied hypergravity (10 x g) caused no significant cell death in NK cells.


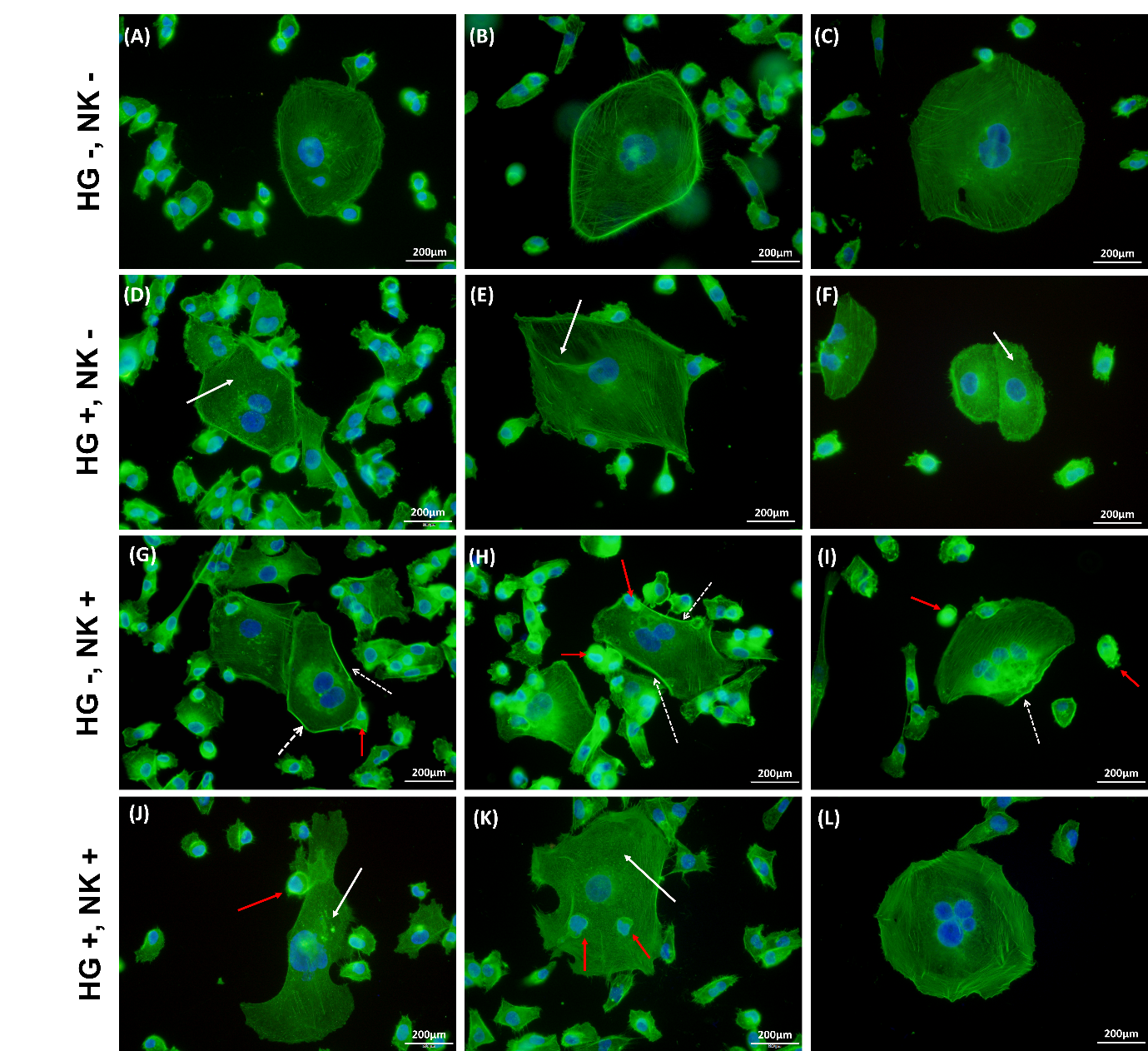


**Supplementary Figure 2.** Full images of F-actin immunofluorescence with Phalloidin in MDA-MB-231. Control group in MDA-MB-231 without hypergravity and without NK cells (A-C); Cell group in MDA-MB-231 with hypergravity and without NK cells (D-F); Cell group in MDA-MB-231 without hypergravity and with NK cells (G-I); Cell group in MDA-MB-231 with both hypergravity and NK cells (J-L). Changes in cytoskeletal structure are indicated with white arrow. The part represented by the red arrow is considered to represent NK cells.


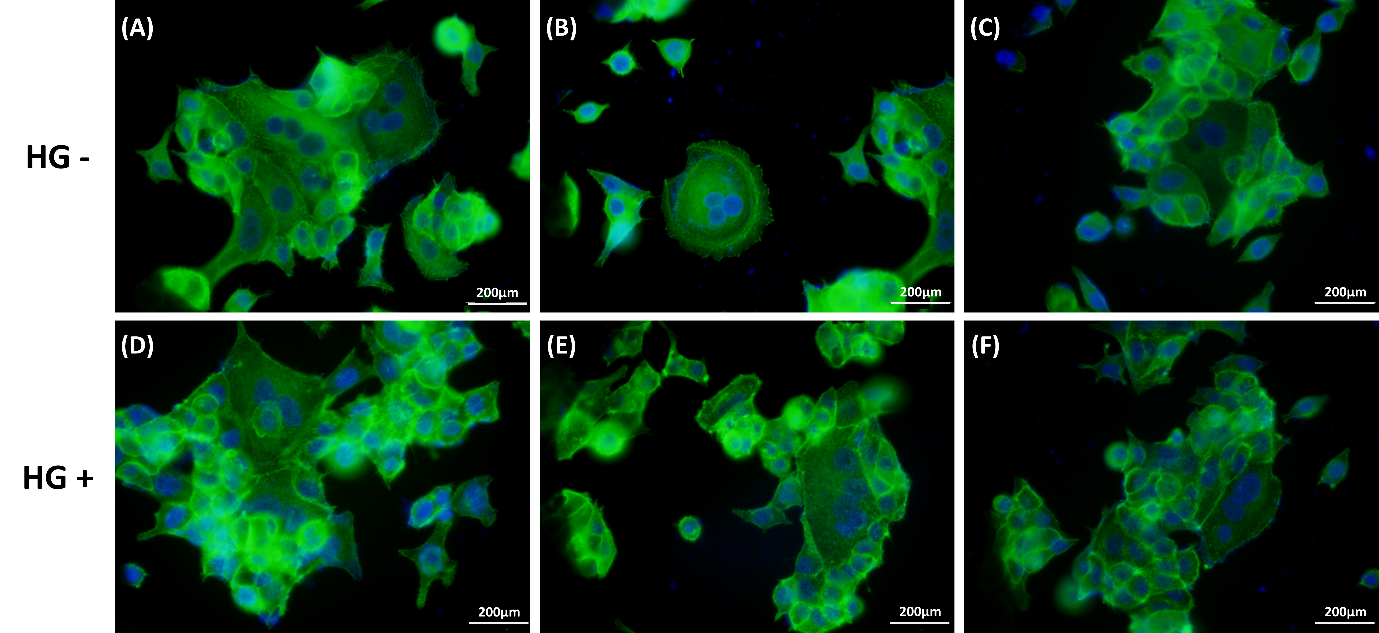


**Supplementary Figure 3.** Immunostaining images of F-actin in MCF-7. Immunofluorescence images of F-actin with AlexaFluor 488 conjugated Phalloidin. Nucleus was counterstained with DAPI. Images of actin response negative control cell line MCF-7: Control group (A-C); Hypergravity treated group (D-F). The applied hypergravity (10 x g) causes no significant change in the cytoskeleton structure of MCF-7 cells.


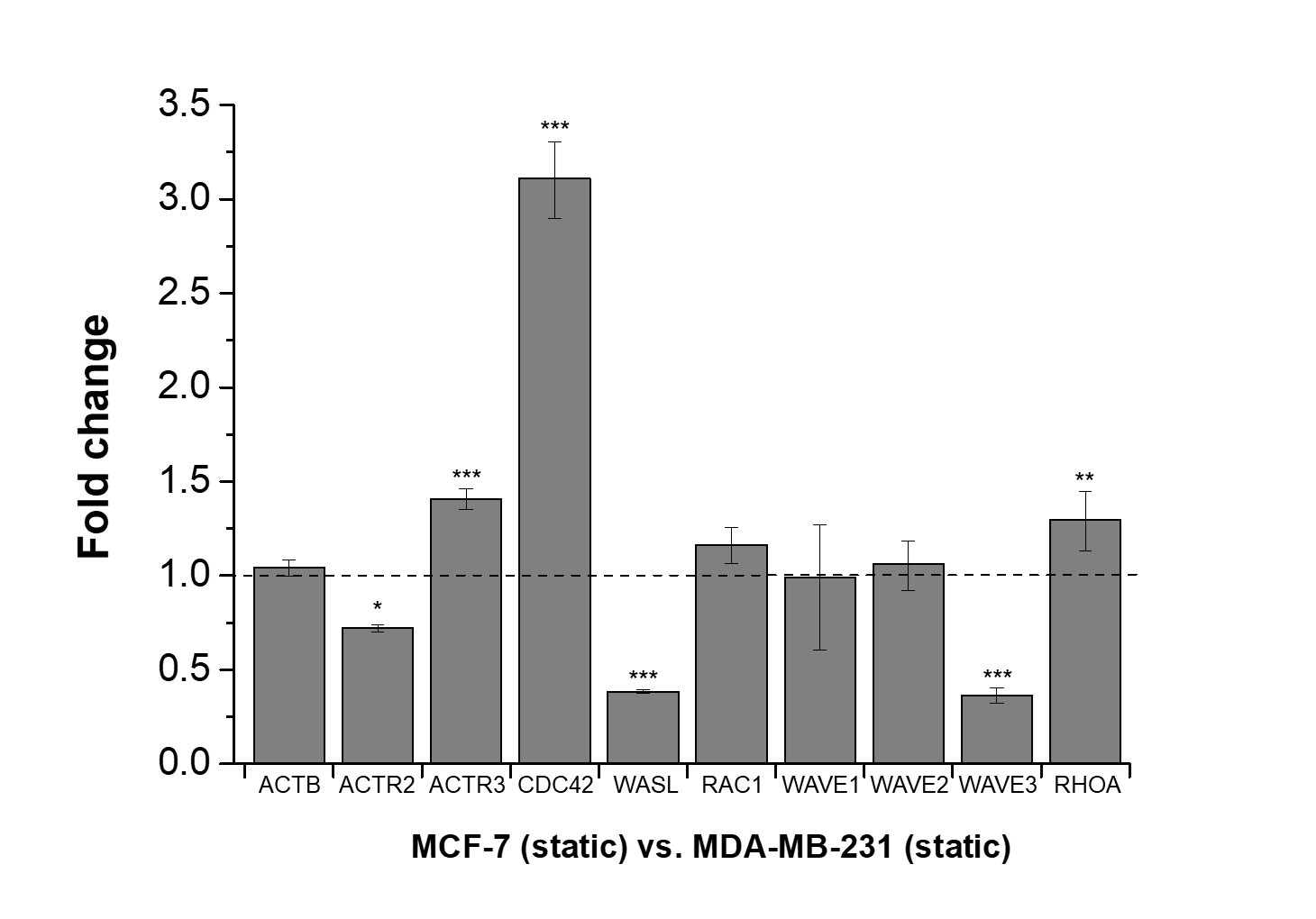


**Supplementary Figure 4.** mRNA expression levels of MDA-MB-231 cells (AR positive) relative to that of MCF-7 cells (AR negative). Relative gene expression levels were calculated using the 2(2DDCT) method. **P*< 0.05, ***P*< 0.01, ****P*< 0.001, compared to MCF-7 cells.


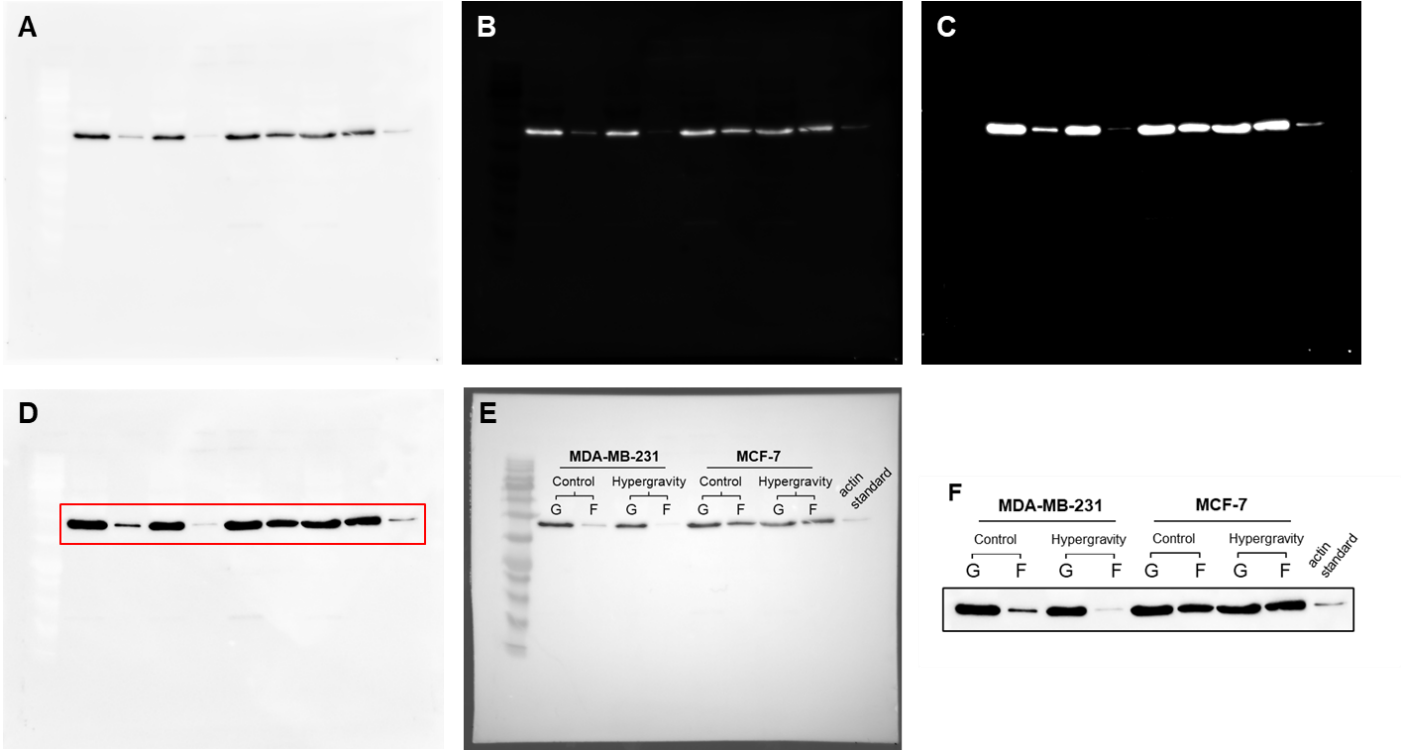


**Supplementary Figure 5.** Full length images of western blots presented in the manuscript. (A) Full length image of actin protein of each F/G-actin sample; (B), (C) inverted and overexposed version of (A) to confirm the borderline of polyvinylidene difluoride (PVDF) membrane; (D) overexposed version of (A); (E) western blot full image of F/G-actin; (F) cropped version (referred to Fig. 3A in manuscript) of (D) with actins standard. Red lines show the cropping locations.
